# Coping strategies adopted by frontline nurses in dealing with COVID‐19 patients in a developing country during the pandemic: A qualitative study

**DOI:** 10.1002/nop2.1614

**Published:** 2023-01-31

**Authors:** Moustaq Karim Khan Rony, Muhammad Mostafijur Rahman, Md. Abdullah Al Saki, Mst. Rina Parvin, Hasnat M. Alamgir

**Affiliations:** ^1^ Master of Public Health Bangladesh Open University Dhaka Bangladesh; ^2^ Institute of Social Welfare and Research University of Dhaka Dhaka Bangladesh; ^3^ Directorate General Nursing and Midwifery Dhaka Bangladesh; ^4^ Textile Engineering College Begumgonj, Noakhali Bangladesh; ^5^ Major at Bangladesh Army Combined Military Hospital Dhaka Bangladesh; ^6^ Professor of Public Health; Chair, Centre for Consultancy and Applied Research International University of Business Agriculture and Technology Dhaka Bangladesh

**Keywords:** coping strategies, COVID‐19, healthcare, nurses, pandemic, patient care

## Abstract

**Aim:**

This study aimed to explore the coping strategies adopted by frontline nurses in dealing with COVID‐19 patients during the pandemic in Bangladesh.

**Design:**

A qualitative descriptive study.

**Methods:**

Purposive sampling was used to recruit seventeen frontline nurses from three COVID‐19‐specific hospitals in Dhaka City. In‐depth online interviews and semi‐structured questionnaires were used to collect data through the Google Meet platform. Interview sessions audio–video were recorded, interpreted, analysed, verbatim transcribed and quotes of the participants were verified by member checking. Thematic analysis was used in this research. The study's reporting guidelines were based on the consolidated criteria for reporting qualitative research.

**Results:**

Seven themes were identified after careful data analysis: (i) A positive attitude in dealing with challenging situation, (ii) Intimate partner's influence, (iii) Self‐emotional regulation, (iv) The tendency to avoid negativity, (v) Motivated by professional obligations, (vi) Religious influence, (vii) Recreational activities.

**No patient or public contribution:**

This study explored various coping strategies employed by frontline nurses in caring for COVID‐19 patients. No patient or public contribution was investigated.

## 1 INTRODUCTION

In recent memory, the coronavirus pandemic has been one of the most fatal of all disease outbreaks. The virus plunged humanity into one of the worst healthcare crises in history (Sarkar et al., 2021). In many parts of the world, the impact of this COVID‐19 pandemic is still ongoing. No one could imagine this happening in the modern era of extensive medical advancement. However, healthcare providers had to deal with this challenging situation and make many personal sacrifices to manage this pandemic. The deadly COVID‐19 virus originated in the Chinese city of Wuhan. The virus infected 52.89 million people worldwide, and 6.30 million died (Worldometer, 2022). Healthcare workers, the frontline fighters, were confronted with the most deadly COVID‐19 virus as they cared for patients, and between 80,000 and 180,000 health workers are estimated to have died (World Health Organization, 2021).

Coping strategies are behavioural and cognitive techniques used to manage stressful situations and factors related to the quality of service provided while rendering clinical responsibilities (Usman & Fahy, 2021). Frontline nurses employed team cohesion, emotional confidence, behavioural control and psychological empowerment as coping mechanisms at work (Bayuo & Agbenorku, 2018). Effective coping strategies significantly influence an individual's improved physical and mental health outcomes (Budimir et al., 2021).

However, nurses used various coping strategies in dealing with COVID‐19 patients, including nurses' self‐strategies (self‐emotional regulation, empathy for patients, self‐protection and recreational activities; Huang et al., 2020), nursing strategies at the ethical level (application of nursing knowledge, attitudes and values, following an evidence‐based practice.), employers' techniques (problem‐focused coping, such as effective planning and sufficient instrumental support, and skill mix: the combination of experienced and new nurses), and nursing leaders' strategies (fostering strong teamwork among nurses; Catania et al., 2021).

During this pandemic, healthcare workers were emotionally and physically harmed due to working in inadequately prepared clinical environments. Denning et al. (2021) revealed that a statistically significant portion of the UK's healthcare workforce was emotionally exhausted from combating COVID‐19 patients. Ayar et al. (2022) also highlighted that healthcare professionals had struggled with work–life balance, depression and mental health issues. Moreover, throughout this pandemic, workplace violence against healthcare workers increased (Zhu et al., 2022). In the USA, 31% of healthcare workers were subjected to abusive behaviour by patients (Akther, 2021). According to a study from Canada, 75% of healthcare personnel were victims of workplace violence during this pandemic (Cregan & Kelloway, 2021). These problems worsened in low‐ and middle‐income countries (LMIC) because they did not have enough healthcare facilities, staffing, planning and clinical equipment (e.g., personal protective equipment, ventilator support; Rony et al., 2021).

Since Bangladesh is an LMIC with a very high population density, the COVID‐19 situation deteriorated here due to the scarcity of healthcare personnel and the small number of healthcare facilities already struggling to cope with increased demand for services (Hossain et al., 2021). Siddiqui (2020) mentioned that the country faced a 0.3 million‐strong healthcare personnel deficit. However, nurses played a vital role in managing patients in the healthcare system because they worked closely with patients and spent most of their time at the patient's bedside. Previous studies investigated a limited number of variables on coping strategies that influenced frontline nurses to care for COVID‐19 patients (Canestrari et al., 2021; Munawar & Choudhry, 2021; Özçevik Subaşi et al., 2021; Zhang, Jiang, et al., 2021; Zhang, Niu, et al., 2021). According to the authors' knowledge and review of literature, no research has been conducted in Bangladesh to learn what drove frontline nurses to care for COVID‐19 patients despite having scarce resources, inadequate healthcare facilities, heavy workloads while the risk of spreading the virus was so high. Therefore, knowing how nurses coped in the clinical settings while caring for COVID‐19 patients is crucial in a resource‐poor setting. This study aimed to determine what coping strategies motivated Bangladeshi frontline nurses in dealing with COVID‐19 patients during the pandemic.

## 2 METHODS

### 2.1 Study design

A descriptive qualitative approach was used to explore the coping strategies that motivated frontline nurses in caring for COVID‐19 patients. This approach was employed because it represents a realistic view of healthcare workers in a healthcare setting (Mulhall, 2003). This study was initiated in three dedicated COVID‐19 hospitals in Dhaka, Bangladesh. The findings of this study were presented using the Consolidated Criteria for Reporting Qualitative Research Checklist (Tong et al., 2007).

### 2.2 Participants

Participants were recruited by purposive sampling from multiple COVID‐19‐specific hospitals in Dhaka City. The following criteria were used to choose participants: (i) have at least 1 year of experience working with COVID‐19 patients. (ii) have worked in a healthcare facility with a capacity of 200 or more patients. (iii) be at least 23 years old.

One co‐author was a frontline nurse who participated in the study because he met the inclusion criteria. As a result, to avoid bias, the co‐author was not involved in the data collection process. Before data collection, investigators provided detailed information about the data collection procedure, potential risks and participant rights. The participants' availability determined the interview schedule.

In this study, the sample size was estimated using the data saturation process. It indicates the most effective coping mechanisms revealed by frontline warriors exposed to COVID‐19 in challenging medical settings. Data saturation was reached until new coping techniques were identified from the participants (Chew et al., 2021; Coyne & Cowley, 2006). Two more participants were interviewed to verify data saturation. The characteristics of participants are represented in Table 1.

**TABLE 1:** Characteristics of participants

No.	UID	Sex	Age (years)	Religion	Marital status	Work experience (years)	Employer type	Job type	Education	Designation	Coping strategies adopted by the participants
R1	3401	F	29	I	Yes	6	Private	T	DNSM	SSN	Self‐emotional regulation, Religious influence
R2	3402	F	34	I	Yes	11	Government	P	MPH	NI	Intimate partners influence, Motivated by professional obligations
R3	3403	M	25	I	No	2	Government	P	BSN	SSN	Motivated by professional obligations, Tendency to avoid negativity
R4	3404	F	41	H	Divorced	17	Government	P	MPH	NI	Self‐emotional regulation, A positive attitude in dealing with challenging situation, Recreational activities
R5	3405	F	38	I	Yes	15	Government	P	DNSM	NI	Tendency to avoid negativity, Recreational activities, Religious influence
R6	3406	F	33.5	I	Yes	10	Private	P	DNSM	SSN	Intimate partners influence, A positive attitude in dealing with challenging situation
R7	3407	M	28	I	No	4.5	Private	T	BSN	SSN	Motivated by professional obligations, Religious influence
R8	3408	F	30	I	Yes	6	Government	P	DNSM	SSN	Tendency to avoid negativity, Recreational activities, Religious influence
R9	3409	M	29.5	H	Yes	6.5	Government	P	DNSM	SSN	Intimate partners influence, Self‐emotional regulation
R10	3410	F	33	I	No	9	Government	T	BSN	SSN	Tendency to avoid negativity, Motivated by professional obligations
R11	3411	F	41	C	Yes	16	Government	P	MPH	NI	Religious influence, Recreational activities
R12	3412	F	28.5	I	Yes	4.5	Government	P	DNSM	SSN	Self‐emotional regulation, A positive attitude in dealing with challenging situation
R13	3413	F	26.5	I	No	2.5	Private	T	DNSM	SSN	Intimate partners influence, Religious influence
R14	3414	F	32	H	Yes	6	Government	P	BSN	SSN	Intimate partners influence, Motivated by professional obligations
R15	3415	F	35	I	Yes	10.5	Government	T	MPH	SSN	Self‐emotional regulation, Tendency to avoid negativity
R16	3416	F	27.5	I	Yes	4	Government	P	BSN	SSN	A positive attitude in dealing with challenging situation, Religious influence
R17	3417	M	34	I	Yes	8.5	Government	P	DNSM	SSN	Intimate partners influence, A positive attitude in dealing with challenging situation

Abbreviations: BSN, Bachelor of science in Nursing; C, Christian; DNSM, Diploma in Nursing Science and Midwifery; F, Female; H, Hindu; I, Islam; M, Male; MPH, Masters of Public Health; NI, Nursing Incharge.; P, Permanent; R, Respondents; SSN, Senior Staff Nurse; T, Temporary; UID, Unique Identification Number.

### 2.3 Questionaries development

The validity and reliability of the questionnaire were determined in two phases. In phase‐ I, questions were sent to two public health research experts for review and then were amended based on their feedback. In phase‐ II, pilot research was conducted to clarify respondents' understanding and eliminate any ambiguity. Finally, three open‐ended questions were used to investigate data about how nurses coped in managing COVID‐19 patients in a healthcare setting, including.

- What has your experience been with COVID‐infected patients?
- How have you been inspired to deal with COVID‐19 patients?
- How did you cope with the challenging COVID‐19 situation?

### 2.4 Data collection

Data were collected from three COVID‐19‐specific hospitals in Dhaka between December 2021 and March 2022. One co‐author abstained from data collection to avoid bias because he was a frontline worker and participant in this study. Other authors gathered email addresses from hospital administration and then sent an invitation email to frontline nurses who met the inclusion criteria. If they accepted, respondents were invited to participate in an in‐depth online interview session with semi‐structured questionnaires via the Google Meet platform for 50–60 min. All interview sessions were conducted entirely in Bengali. Audio and video recording of the interviews were done for data interpretation and analysis. The interview session was scheduled at the convenience of the participants.

### 2.5 Data analysis

**FIGURE 1:**
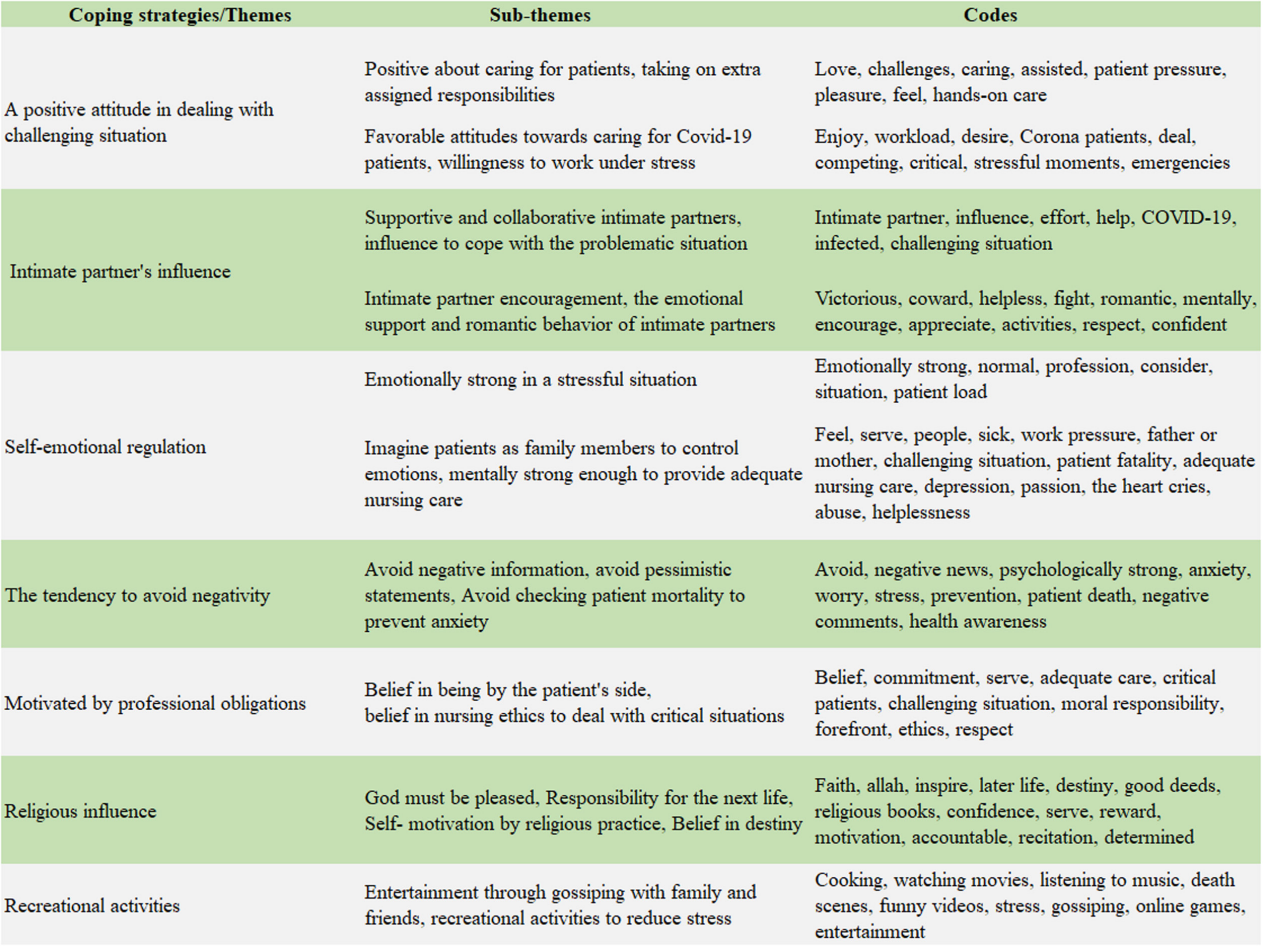
Thematic analysis

Three authors separately listened to recorded interviews multiple times and then transcribed the participant quotes. The authors then mailed each respondent's quotes back to the same respondent to verify data accuracy and internal validity. Participants' acknowledgment and confirmation of the quotes were kept. Irrelevant quotes that did not match the study's objectives were removed. Only data related to the study purpose were kept for the final analysis and translated from Bangla to English. After that, an expert translator reviewed the data set for originality. Secondly, the authors extracted codes from primary data individually. Thirdly, the authors eliminated duplicate codes and then similar codes were separated to form subthemes (Figure 1). Following that, emerging themes were developed. Thematic analysis is a method academics use to find deep insights into a phenomenon and discover the connections between variables (Alhojailan, 2012).

### 2.6 Ethical considerations

The International Nursing College, Bangladesh, review committee approved this study (HRM/INC/319/1/11/2021). Before the interview began, participants were informed about the right to withdraw from the research and that their voluntary nature of participation. In addition, participants' scanned signatures were obtained through email prior to conducting the online interviews. Participants were assured that their information would be kept confidential.

### 2.7 Rigour

The trustworthiness of this study was determined based on the following four criteria: credibility, transferability, dependability and confirmability: (i) member checking was used to verify that the primary data collection was credible; (ii) an audit trail was maintained by retaining the original data set's evidence (audio recording and handwritten datasheet); the audit trail demonstrates that the investigation has derived evidence‐based findings; (iii) each author examined the data independently to confirm the study's dependability; (iv) triangulation was used to determine the confirmability of this study's findings; (v) data saturation was achieved to assure transferability.

## 3 RESULTS

### 3.1 Characteristics of the participants

Seventeen respondents participated in this study with the majority (*n* = 13; 76.47%) being female. Their ages ranged from 25 to 41 years (mean = 31.5 years; SD = 3.54). Participants' working experience ranged between 2 and 17 years (mean = 1.77 years; SD = 7.25). Among them, 76.47% (*n* = 13) were government job holders, 23.53% (*n* = 4) were private job holders, 23.53% (*n* = 4) were nursing incharge and 76.47% (*n* = 13) were senior staff nurses. With respect to education, 23.53% (*n* = 4) obtained master's degrees, 29.41% (*n* = 5) completed Bachelor of Science in Nursing degrees, and 47.06% (*n* = 8) completed Diploma in Nursing and Midwifery degrees. Twelve nurses were married and four were unmarried while one was divorced. In term of religious belief, 13 nurses were Muslim, three were Hindu, and one was Christian.

### 3.2 Thematic analysis

Seven themes were identified from the data analysis that described how frontline nurses coped with the COVID‐19 pandemic while caring for the coronavirus‐infected patients.

#### 3.2.1 A positive attitude in dealing with challenging situation

A positive interest in work increases positive emotions, morale and job satisfaction. In contrast, negative contacts at work produce misunderstandings, worry, tension and anxiety, which have a negative impact on the working environment and decrease organizational productivity. Nurses were positive about caring for patients, especially in hands‐on care, supporting colleagues, handling new challenging situations and taking on extra responsibilities to ensure nursing care quality.

If the nurses noticed that one coworker's workload was excessive, the other nurses assisted in caring for their COVID‐19 patient; I think we love to take on challenges” (R17, male, senior staff nurse, 8.5 years of working experience).

The patient pressure in the hospital was too much, so I would go to the workplace an hour earlier and get out too late. However, I feel good about hands‐on care (R6, female, senior staff nurse, 10 years of working experience)

Optimistic nurses are supposed to give idealistic service, empathetic care for patients, and confidence in protecting factors related to clinical problems. Nurses exhibited a favourable attitude toward caring for COVID‐19 patients and a willingness to work under stress.

Sometimes, I refused to take weekends off due to the high workload at the hospital; I do not like to enjoy my time when my colleagues are under stress. (R12, female, senior staff nurse, 4.5 years of working experience)

I have a strong desire to work with Corona patients. I want to deal with critical clinical situations and keep myself calm when handling stressful moments (R4, female, nursing incharge, 17 years of working experience)

The nurses expressed great enthusiasm for the patient's work, and I once saw two nurses competing, one asking the other nurse to rest so she could handle the patient” (R16, female, senior staff nurse, 4 years of working experience)

#### 3.2.2 Intimate partners influence

Intimate partners' encouragement helps the working partner reach goals by assisting them in keeping track of the right path and offering much‐needed emotional and mental support. The nurses' private partners were supportive and collaborative in general, and their assistance provided the nurses with the strength they required to cope with the difficult situation they faced.

My intimate partner influenced me to do my best, even though I hadn't seen him in nine months. I promised him that I would make every effort to provide the highest quality nursing care (R13, female, senior staff nurse, 2.5 years of working experience)

Because of the nursing shortage, nurses were subjected to high levels of stress at their places of employment. Some nurses wanted to leave their jobs, but their intimate partners encouraged them to assist COVID‐19 patients.

In the words of my intimate partner…if you love me, you must help the COVID‐19 infected patients (R9, male, senior staff nurse, 6.5 years of working experience)

Please do not come to me like a coward, abandoning helpless patients infected with the coronavirus. (R17, male, senior staff nurse, 8.5 years of working experience)

Nursing is challenging since it deals with human life. In a healthcare setting in a developing nation, it is difficult for nurses to maintain a healthy work–life balance due to the high workload. Nevertheless, a supportive partner is necessary for maintaining a healthy work–life balance. During the pandemic, intimate partners encouraged nurses to provide competent nursing care with confidence by providing emotional support and engaging in romantic behaviour.

My intimate partner was my source of motivation to work in the COVID‐19 critical situation. He was mentally supportive, which helped me encourage myself (R2, female, nursing incharge, 11 years of working experience)

My husband always appreciated all my activities, which I had never noticed before (R6, female, senior staff nurse, 10 years of working experience)

My relationship gave me the emotional fortitude to care for infected people during that period. (R14, female, senior staff nurse, 6 years of working experience)

#### 3.2.3 Self‐emotional regulation

Maintaining one's efficacy in the face of adversity requires emotional self‐control that allows one to rein in disruptive thoughts and feelings. During this COVID‐19 pandemic, nurses were able to deal with stressful situations by controlling their emotions. Nurses worked long shifts regularly, went on working for months without taking a vacation, and battled against COVID‐19.

Due to the heavy patient load, it was hard enough to find time to eat snacks or even go to the bathroom. I had 12 hours of daily duty. The hospital seemed to be my home; I tried to consider that situation as a normal part of my profession to make me emotionally strong (R12, female, senior staff nurse, 4.5 years of working experience)

The ability to feel strong emotions is crucial for providing care to patients. To manage critical situations, nurses must develop the ability to regulate emotions and act responsibly. Inadequacy in emotional control can result in adverse patient outcomes. Nurses who could not spend time with their families during this COVID‐19 pandemic controlled their emotions by imagining patients as family members.

My father was sick in that challenging situation. Due to work pressure and lockdown, I could not go home. I had to serve Corona patients in the hospital. However, I felt that my serving of people were just like serving my father or mother (R1, female, senior staff nurse, 6 years of working experience)

My child had severe pneumonia. My heart was crying for him, but I had no vacation. Our responsibility was to return to the workplace immediately after quarantine. I controlled my emotions by just thinking that my family members were taking care of her. But who will care for them (patients) if I leave here? (R15, female, senior staff nurse, 10.5 years of working experience)

Strong emotional regulation abilities can also improve the long‐term health and well‐being of employees, enhancing their performance at work and therapeutic interactions in healthcare settings. Despite feeling unhappy and abused due to the extra workload, the nurses were emotionally strong enough to give adequate nursing care.

Because of the high patient fatality rate and patient load, I was depressed. When I used to go to the hospital, my chest throbbed. On the other hand, the cries of the patient's relatives were floating in my eyes. Then I consoled myself by telling myself that my passion should only be for them (patients) (R9, male, senior staff nurse, 6.5 years of working experience)

Sometimes the abuse of the patient and the patient's family members makes my heart cry, but I comfort my mind by saying that their abuse is nothing compared to their helplessness (R4, female, nursing incharge, 17 years of working experience)

#### 3.2.4 The tendency to avoid negativity

A positive outlook people can deal with a traumatic situation by keeping a positive frame of mind. When faced with adversity, they rise to the purpose rather than giving up by avoiding negativity. During this pandemic, nurses avoided negative information about COVID‐19 in dealing with the situation.

When using Facebook, YouTube, or other online platforms, I avoided negative news about COVID‐19 to keep me psychologically strong (R3, male, senior staff nurse, 2 years of working experience)

Yam, yes, I heard minimal news about COVID‐19 for stress prevention (R5, female, nursing incharge, 15 years of working experience)

Avoiding negative information aids in maintaining mental confidence and enhancing work performance. It boosts the worker's energy levels, keeping them awake and make them ready to do their specific tasks. Positive thinking helps enhance the quality of care by reducing stress. To stay mentally strong in this pandemic, nurses were also conscientious about avoiding pessimistic statements.

If anyone around me spoke negatively about the pandemic, I avoided that negativity to reduce tension (R10, female, senior staff nurse, 9 years of working experience)

If anyone commented negatively about the pandemic situation, instead of responding to their views, I underlined the necessity of boosting health awareness to minimize COVID‐19 contamination (R15, female, senior staff nurse, 10.5 years of working experience)

Avoiding negative occurrences could help reduce anxiety, sadness, tension and low self‐esteem, which boosts mental confidence at work. When the patient mortality rate in the hospital was extremely high, the nurses avoided checking the patient mortality rate to prevent anxiety related to COVID‐19 infection.

I avoided checking patient death records in the hospital's computer system so that I did not get worried by the high mortality rate of patients (R8, female, senior staff nurse, 6 years of working experience)

#### 3.2.5 Motivated by professional obligations

In a nutshell, a nurse's professional obligations describe the most fundamental things related to nursing practice and the objectives of nursing. It helps shape one's career as a nurse and points one toward the areas of expertise. In this study, nurses were committed to their belief that, as nurses, they should always be able to care for patients, which encouraged them to give comprehensive care during the pandemic circumstances.

As a nurse, I believe I should not leave critically ill patients; I should serve them no matter how bad the situation gets (R3, male, senior staff nurse, 2 years of working experience)

Ever since I took the oath as a nurse, I have always believed that I would be able to take care of sick people in any situation (R7, male, senior staff nurse, 4.5 years of working experience)

Nurses' professional obligations are crucial for facilitating interactions between patients, attendants and other caregivers. It motivates nurses to practice competently, ethically and scientifically. In this study, faith in nursing ethics did inspire nurses to adapt to challenging clinical settings to give effective nursing care.

I think, as a nurse, my moral responsibility is to always be at the forefront in dealing with patients (R2, female, nursing incharge, 11 years of working experience)

From my ethics, I could not deny any challenging situations; I have to manage these (R14, female, senior staff nurse, 6 years of working experience)

Hmm… It is my responsibility to respect patients and provide adequate care for their healing when needed (R10, female, senior staff nurse, 9 years of working experience)

#### 3.2.6 Religious influence

The religious practice contributes to greater self‐esteem and psychological health. Belief in a religion may improve a person's disposition and confidence to assume obligations appropriately. Nurses believe that God is incharge of everything and that the most excellent way to please Him is to serve patients with COVID‐19.

I thought, what will I answer to Allah after death if I don't stand by these sick people now? (R7, male, senior staff nurse, 4.5 years of working experience)

I am inspired to serve them because the prayers of these sick people will help me in my later life (R11, female, nursing incharge, 16 years of working experience)

Religion could be a source of strength for persons facing adversity, whether it is a physical ailment, overload, or unhappiness. Those who turn to their faith in times of need seem to fare better than those who are not. In this COVID‐19 pandemic, nurses were further motivated to fight by embracing the belief that if they helped COVID‐19 patients, they would be rewarded in later life, and if they did not, they would be held accountable for their negligence.

It is my duty to go ahead as a frontline fighter in any situation. God sees everything. I have to be held accountable if I can't give my best (R16, female, senior staff nurse, 4 years of working experience)

There is a better reward for good deeds in the next life, and what could be better than standing by a sick person? (R1, female, senior staff nurse, 6 years of working experience)

Religious people are better able to handle stress and despair and are less likely to be affected by these. It is possible that engaging in particular religious rituals can cause brain changes that benefit mental health. Similarly, nurses practiced their religion to increase their self‐motivation to cope with the devastating pandemic crisis.

When I got anxious because of COVID‐19 fear and work stress, I used to recite the Qur'an, read religious books, and pray to boost my confidence (R13, female, senior staff nurse, 2.5 years of working experience)

An individual's religious beliefs may benefit their loyalty to the organization, sense of morale and ability to interact with co‐workers, all of which contribute to enhanced productivity. Furthermore, the incorporation of religious elements improved workers' resilience to stress in the workplace. Belief in fate inspired nurses to deal with infected COVID‐19 patients.

I believe that whatever Allah puts into your destiny will happen; I have to serve the people (R5, female, nursing incharge, 15 years of working experience)

God determined my destiny before I was born, so I am not afraid to serve patients, but yes, I believe my good deeds will change my destiny (R8, female, senior staff nurse, 6 years of working experience)

#### 3.2.7 Recreational activities

Participating in recreational activities has been related to improved mental health, higher self‐confidence and independence, a more fertile imagination and a more satisfying sense of accomplishment. We explored that nurses used various recreational activities to reduce stress because their leave was limited, and going out was restricted due to lockdown.

I cooked new items to reduce stress, but occasionally watched movies and listened to music (R5, female, nursing incharge, 15 years of working experience)

I made some funny short videos to get rid of the corona patient's death scene floating in my mind (R8, female, senior staff nurse, 6 years of working experience)

Reducing fatigue and exhaustion via recreation is a great way to unwind. Executing the same task or activity repeatedly and continuously for an extended period causes stress and burnout. Throughout the outbreak, nurses kept themselves entertained by sharing their emotions with their favourite people and playing online games.

When I was stressed, I amused myself by gossiping with my relatives and friends through Messenger and WhatsApp (R4, female, nursing incharge, 17 years of working experience)

Smiling…I played online games to keep my mind up (R11, female, nursing incharge, 16 years of working experience).

## 4 DISCUSSION

Due to the unprecedented number of coronavirus infection cases during the pandemic, medical facilities worldwide had a difficult time treating infected individuals. This study investigated how frontline nurses dealt with COVID‐19‐infected patients during that challenging clinical situation using various coping strategies. We revealed that nurses were able to tackle the pandemic issue from their positive enthusiasm in dealing with the challenging situation created. Nursing intervention is efficiently accomplished when nurses have a positive interest about their workplace (Dobrowolska et al., 2021; Jiang et al., 2021). Positive interest at work results in higher levels of service quality (Othman & Nasurdin, 2019). According to Kim‐Soon et al. (2022), there is a strong link between nurses' positive interest in nursing care and patient satisfaction. Because a positive attitude assists nurses in adjusting to difficult situations by eliminating factors associated with an adverse working environment (Kim et al., 2018), Badu et al. (2020) also discussed that nurses' positive interest in performing clinical responsibilities is one of the most critical tactics in dealing with workload situations that encourage nurses to engage in hands‐on care. Moreover, the positive patient outcomes in healthcare organizations largely depend on nurses' positive attitudes toward patient management (Nashwan et al., 2021). Several studies have demonstrated that a nurse's negative attitude significantly affects providing appropriate nursing care (Rayner et al., 2019; Weare et al., 2019). However, developing coping strategies enhances the nurses' interest in handling critically ill patients (Jiang et al., 2021).

During this pandemic, the influence of intimate partners of nurses contributes as a source of coping strategies to adjust to the stressful hospital environment. Intimate partners' support helps healthcare workers feel more confident mentally, which allows them to avoid emotional distress (Sundborg et al., 2018). Supportive intimate partners considerably contribute to constructing a healthy work–life balance (Jack et al., 2019), positive feelings about one's job (Alvarez et al., 2018), higher‐quality service (Ogbe et al., 2020) and psychological well‐being (De Kock et al., 2021), all of which boost nurses to manage a stressful working condition (McLindon et al., 2018). Employees who receive adequate support from an intimate partner are more likely to engage in high‐quality work (Keynejad et al., 2020). Additionally, the positive influence of an intimate partner might be beneficial in maintaining one's determination, managing work–family conflict and engaging efficiently at work (Bermele et al., 2018). In contrast, the negative impact of an intimate partner creates poor attention at work (Anderson et al., 2018), job burnout and dissatisfaction, which lessens job interest and makes it more challenging to continue the job (Deen et al., 2022).

The current study revealed that nurses could maintain self‐control while caring for COVID‐19 patients amid the terrible pandemic. Emotional self‐control enables nurses to handle their job workloads better and develop self‐confidence and self‐resilience (Hatami et al., 2022; Rony et al., 2022). It can help resolve work‐related conflicts by developing work engagement, empathy for patients and passion for nursing tasks in the clinical setting (Cuartero & Tur, 2021). This coping technique also develops nurses' ability to make proper decisions in the healthcare setting and sustains nurses' emotional adjustment by recognizing unpleasant feelings associated with working circumstances (Cooper, 2018). Bahrami et al. (2018) demonstrated that emotional self‐control is crucial for maintaining a healthy emotional balance to give adequate nursing care. Furthermore, maintaining one's efficacy under trying or even hostile circumstances requires a certain degree of emotional self‐control. As healthcare providers, nurses should develop emotional control, display good traits such as empathy and compassion, and avoid negative things such as annoyance, hostility and anxiety (Lee & Jang, 2019).

During the COVID‐19 situation, nurses ignored negativity related to COVID‐19 news that dramatically impacted their ability to become mentally strong. Nurses avoided negative news about COVID‐19 to avoid suffering from psychological distress. This coping mechanism helps nurses handle the stresses of caring for critically suffering patients (Besirli, 2020). Cho and Steege (2021) investigated that avoiding negative things impacts nursing performance and can improve nurses' emotional well‐being and professional commitment. Furthermore, avoiding negativity increases job happiness, job retention and productivity, with positive patient outcomes (Rasool et al., 2021). In contrast, if healthcare employees receive unfavourable information related to the workplace, this can depress their minds, leading to feelings of patient burden during patient care (Richards et al., 2022). Additionally, experiencing negative factors about working conditions impacts the reduction of mental strength (Zhang, Jiang, et al., 2021; Zhang, Niu, et al., 2021).

The current study explored nurses' professional obligations when dealing with COVID‐19 patients during the pandemic. Nursing professional obligations embody nurses' ethics, values and motivations to give excellence in care for the patients (Ghazanfari et al., 2022; Suandika et al., 2021). It aids nurses in understanding professional objectives and nursing principles (McNett et al., 2021). Pajakoski et al. (2021) remarked that nursing professional obligations help nurses manage a less‐than‐ideal working environment and deliver better patient health outcomes. This coping strategy improves the problem‐solving ability of nurses, influencing them to give higher‐quality nursing care (Bayram et al., 2022). Furthermore, nursing professional obligations strengthen nurses' commitment to caring for critical patients (Adam et al., 2021). On the other hand, nurses' insufficient professional obligations suppressed their enthusiasm for patient bedside practice (Sapri et al., 2022).

Moreover, frontline nurses were particularly encouraged by their religious beliefs when caring for COVID‐19 patients. Nursing practice must be coherent and internally consistent with religious beliefs and practices (Badanta et al., 2021). There could be religious or spiritual distress if one's religious views and treatment plans do not match up (Registered Nursing, 2022). Combining religious beliefs and professional obligations results in a substantial adjustment at work, leading to more beneficial service outcomes (de Diego‐cordero et al., 2022). Rathnayake et al. (2021) suggested that religious influence is an efficacious mental stress coping mechanism that drives nurses' belief to do their best nursing care service.

This study also investigated that nurses were influenced to participate in caring for COVID‐19‐infected patients amid the catastrophic pandemic by doing various recreational activities. Leisure activities help minimize work‐related stress and improve mental refreshment, capable nurses to maintain their stamina when doing nursing responsibilities in challenging circumstances (Wolff et al., 2021). In addition, engaging in leisure activities is essential to enhancing the quality of life that reduces mental weariness, lessens exhaustion, facilitates adjusting to stressful work environments and boosts productivity (Faulkner & Biddle, 2002; Yektatalab et al., 2022). Pinho et al. (2021) highlighted that engaging in leisure activities might assist people in forgetting about their stressors and encouraging their minds to function more efficiently. Overall, a healthcare worker's mental health might benefit immensely by adopting certain coping strategies and thereby improve organization's expected outcomes.

### 4.1 Implications of this study

This study's findings will aid nurses in adjusting to the workload, fostering positive psychological outcomes and enhance emotional self‐control. This study encourages frontline fighters to deal with difficult situations and become more resilient. This study findings elaborated on how frontline nurses adopted various coping strategies to offer quality patient care in contested situations. It also revealed how coping skills helped nurses avoid occupational stressors and maintain a healthy work–life balance. This study suggests healthcare workers acquire coping skills to deliver high‐quality medical service. In addition, it suggests that healthcare institutions implement training programs in coping skills adoption. These techniques would assist nurses in dealing with challenging clinical conditions and providing high‐quality nursing care. Finally, this study suggests conducting additional quantitative research using the coping scale to investigate nurses' effective coping techniques. This would help to identify the predictor variables that positively influence adopting coping strategies.

### 4.2 Limitations and recommendations

In this study, an online interview was conducted with frontline nurses due to the COVID‐19 protection limits and the convenience of frontline nurses. A few participants were private job holders. So, it is essential to conduct other studies on a large scale to find out the coping strategies adopted by frontline nurses working in the private job sector. In addition, to find more effective coping mechanisms, a qualitative study should be conducted with nurse managers and leaders who led frontline nurses throughout the COVID‐19 pandemic. This would contribute to exploring different inspirational and survival strategies for nurses. It is recommended other studies be conducted to develop a coping scale instrument to assess the level of coping skills adopted by frontline nurses, which would aid in examining the relationship between the quality of nursing care and the use of coping mechanisms.

## 5 CONCLUSIONS

Coping strategies help nurses have emotional self‐control and give effective nursing care. The quality of patient care is highly dependent on how well a nurse can adopt their coping techniques to adapt to working environments. Coping strategies enable nurses deal with difficult situations and develop greater resilience in their workplace, particularly in resource‐poor settings. Coping skills also assist nurses in maintaining psychological well‐being and dealing with stressful working conditions. Moreover, coping mechanisms strongly influence nurses' ability to maintain a relatively healthy work–life balance.

## AUTHORS' CONTRIBUTION

MKKR conceptualized the study. MKKR and MRP involved in formal analysis. MKKR, MMR, MRP and HMA provided the methodology. MKKR, MMR, MAAS and HMA wrote the original draft. MKKR, MRP, MAAS and HMA reviewed and edited. All authors have read and approved the final version of the manuscript. We had full access to all of the data in this study and takes complete responsibility for the integrity of the data and the accuracy of the data analysis.

## FUNDING INFORMATION

There was no external fund taken for this current research.

## CONFLICT OF INTEREST STATEMENT

The authors' have no competing interest at all.

## ETHICAL APPROVAL

The International Nursing College, Bangladesh, review committee approved this study (HRM/INC/319/1/11/2021). Before the interview began, participants were informed about the right to withdraw from the research and that their voluntary nature of participation. In addition, participants' scanned signatures were obtained through email prior to conducting the online interviews. Participants were assured that their information would be kept confidential.

## Data Availability

The data sets used and analysed during the current study are available on reasonable request.

## References

[nop21614-bib-0001] Adam, S. , Juergensen, L. , & Mallette, C. (2021). Harnessing the power to bridge different worlds: An introduction to posthumanism as a philosophical perspective for the discipline. Nursing Philosophy, 22(3), e12362. 10.1111/nup.12362 34157215

[nop21614-bib-0002] Akther, A. (2021). Nurses say patients are getting more abusive, and simple questions can set them off. Retrieved from https://www.businessinsider.com/frontline‐registered‐nurses‐see‐rise‐in‐physical‐and‐verbal‐abuse‐2021‐10

[nop21614-bib-0003] Alhojailan, M. I. (2012). Thematic analysis: A critical review of its process and evaluation. West East Journal of Social Sciences, 1(1), 39–47.

[nop21614-bib-0004] Alvarez, C. , Debnam, K. , Clough, A. , Alexander, K. , & Glass, N. E. (2018). Responding to intimate partner violence: Healthcare providers' current practices and views on integrating a safety decision aid into primary care settings. Research in Nursing & Health, 41(2), 145–155. 10.1002/nur.21853 29441596

[nop21614-bib-0005] Anderson, J. C. , Campbell, J. C. , Glass, N. E. , Decker, M. R. , Perrin, N. , & Farley, J. (2018). Impact of intimate partner violence on clinic attendance, viral suppression and CD4 cell count of women living with HIV in an urban clinic setting. AIDS Care, 30(4), 399–408. 10.1080/09540121.2018.1428725 29397777 PMC5830100

[nop21614-bib-0006] Ayar, D. , Karaman, M. A. , & Karaman, R. (2022). Work‐life balance and mental health needs of health professionals during COVID‐19 pandemic in Turkey. International Journal of Mental Health and Addiction, 20(1), 639–655. 10.1007/s11469-021-00717-6 34849106 PMC8612392

[nop21614-bib-0007] Badanta, B. , Rivilla‐García, E. , Lucchetti, G. , & de Diego‐Cordero, R. (2021). The influence of spirituality and religion on critical care nursing: An integrative review. Nursing in Critical Care, 27(3), 348‐366. 10.1111/nicc.12645 33966310

[nop21614-bib-0008] Badu, E. , O'Brien, A. P. , Mitchell, R. , Rubin, M. , James, C. , McNeil, K. , Nguyen, K. , & Giles, M. (2020). Workplace stress and resilience in the Australian nursing workforce: A comprehensive integrative review. International Journal of Mental Health Nursing, 29(1), 5–34. 10.1111/inm.12662 31917519

[nop21614-bib-0009] Bahrami, M. , Purfarzad, Z. , Keshvari, M. , Rafiei, M. , & Sivertsen, N. (2018). Emotional competence: A core competence in gerontological nursing in Iran. International Journal of Older People Nursing, 13(4), e12210. 10.1111/opn.12210 30248241

[nop21614-bib-0010] Bayram, A. , Özsaban, A. , Durgun, H. , Aksoy, F. , Turan, N. , Köktürk Dalcali, B. , & Oksay Şahin, A. (2022). Nursing students' perceptions of nursing diagnoses, critical thinking motivations, and problem‐solving skills during distance learning: A multicentral study. International Journal of Nursing Knowledge, 33(4), 304‐311. 10.1111/2047-3095.12362 35244349

[nop21614-bib-0011] Bayuo, J. , & Agbenorku, P. (2018). Coping strategies among nurses in the burn intensive care unit: A qualitative study. Burns Open, 2(1), 47–52. 10.1016/j.burnso.2017.10.004

[nop21614-bib-0012] Bermele, C. , Andresen, P. A. , & Urbanski, S. (2018). Educating nurses to screen and intervene for intimate partner violence during pregnancy. Nursing for Women's Health, 22(1), 79–86. 10.1016/j.nwh.2017.12.006 29433702

[nop21614-bib-0013] Besirli, A. (2020). The relationship between anxiety and depression levels with perceived stress and coping strategies in health care workers during the COVID‐19 pandemic. SiSli Etfal Hastanesi Tip Bulteni/The Medical Bulletin of Sisli Hospital, 55, 1–11. 10.14744/SEMB.2020.57259 PMC8085458 33935529

[nop21614-bib-0014] Budimir, S. , Probst, T. , & Pieh, C. (2021). Coping strategies and mental health during COVID‐19 lockdown. Journal of Mental Health, 30(2), 156–163. 10.1080/09638237.2021.1875412 33502917

[nop21614-bib-0015] Canestrari, C. , Bongelli, R. , Fermani, A. , Riccioni, I. , Bertolazzi, A. , Muzi, M. , & Burro, R. (2021). Coronavirus disease stress among Italian healthcare workers: The role of coping humor. Frontiers in Psychology, 11, 601574. 10.3389/fpsyg.2020.601574 33569023 PMC7868596

[nop21614-bib-0016] Catania, G. , Zanini, M. , Hayter, M. , Timmins, F. , Dasso, N. , Ottonello, G. , Aleo, G. , Sasso, L. , & Bagnasco, A. (2021). Lessons from Italian front‐line nurses' experiences during the COVID‐19 pandemic: A qualitative descriptive study. Journal of Nursing Management, 29(3), 404–411. 10.1111/jonm.13194 33107657

[nop21614-bib-0017] Chew, Y. J. M. , Ang, S. L. L. , & Shorey, S. (2021). Experiences of new nurses dealing with death in a paediatric setting: A descriptive qualitative study. Journal of Advanced Nursing, 77(1), 343–354. 10.1111/jan.14602 33074568

[nop21614-bib-0018] Cho, H. , & Steege, L. M. (2021). Nurse fatigue and nurse, patient safety, and organizational outcomes: A systematic review. Western Journal of Nursing Research, 43(12), 1157–1168. 10.1177/0193945921990892 33554767

[nop21614-bib-0019] Cooper, C. L. (2018). Managerial, occupational and organizational stress research. https://search.ebscohost.com/login.aspx?direct=true&scope=site&db=nlebk&db=nlabk&AN=1692822

[nop21614-bib-0020] Coyne, I. , & Cowley, S. (2006). Using grounded theory to research parent participation. Journal of Research in Nursing, 11(6), 501–515. 10.1177/1744987106065831

[nop21614-bib-0021] Cregan, B. , & Kelloway, E. K. (2021). Physical intimidation and bullying in the workplace. In P. D'Cruz , E. Noronha , L. Keashly , & S. Tye‐Williams (Eds.), Special topics and particular occupations, professions and sectors (Vol. 4, pp. 33–53). Springer Singapore. 10.1007/978-981-10-5308-5_4

[nop21614-bib-0022] Cuartero, N. , & Tur, A. M. (2021). Emotional intelligence, resilience and personality traits neuroticism and extraversion: Predictive capacity in perceived academic efficacy. Nurse Education Today, 102, 104933. 10.1016/j.nedt.2021.104933 33957394

[nop21614-bib-0023] de Diego‐cordero, R. , López‐Gómez, L. , Lucchetti, G. , & Badanta, B. (2022). Spiritual care in critically ill patients during COVID‐19 pandemic. Nursing Outlook, 70(1), 64–77. 10.1016/j.outlook.2021.06.017 34711420 PMC8226065

[nop21614-bib-0024] De Kock, J. H. , Latham, H. A. , Leslie, S. J. , Grindle, M. , Munoz, S.‐A. , Ellis, L. , Polson, R. , & O'Malley, C. M. (2021). A rapid review of the impact of COVID‐19 on the mental health of healthcare workers: Implications for supporting psychological well‐being. BMC Public Health, 21(1), 104. 10.1186/s12889-020-10070-3 33422039 PMC7794640

[nop21614-bib-0025] Deen, C. M. , He, Y. , Gregg, H. , Restubog, S. L. D. , & O'Leary‐Kelly, A. (2022). Intimate partner aggression and work: An interdisciplinary review and agenda for future research. Journal of Organizational Behavior, 43(2), 236–259. 10.1002/job.2585

[nop21614-bib-0026] Denning, M. , Goh, E. T. , Tan, B. , Kanneganti, A. , Almonte, M. , Scott, A. , Martin, G. , Clarke, J. , Sounderajah, V. , Markar, S. , Przybylowicz, J. , Chan, Y. H. , Sia, C.‐H. , Chua, Y. X. , Sim, K. , Lim, L. , Tan, L. , Tan, M. , Sharma, V. , … Kinross, J. (2021). Determinants of burnout and other aspects of psychological well‐being in healthcare workers during the Covid‐19 pandemic: A multinational cross‐sectional study. PLoS One, 16(4), e0238666. 10.1371/journal.pone.0238666 33861739 PMC8051812

[nop21614-bib-0027] Dobrowolska, B. , Whelan, J. , & Timmins, F. (2021). Managing holistic nursing practice—The need for spiritual care competence in healthcare practice. Journal of Nursing Management, 30, 1083–1086. 10.1111/jonm.13538 34964532

[nop21614-bib-0028] Faulkner, G. , & Biddle, S. (2002). Mental health nursing and the promotion of physical activity. Journal of Psychiatric and Mental Health Nursing, 9(6), 659–665. 10.1046/j.1365-2850.2002.00520.x 12472818

[nop21614-bib-0029] Ghazanfari, M. J. , Esmaeili, S. , Emami Zeydi, A. , & Karkhah, S. (2022). Moral distress among nurses during covid‐19 pandemic: Challenges and coping strategies. Nursing Open, 9, 2227–2228. 10.1002/nop2.1248 35606893 PMC9190694

[nop21614-bib-0030] Hatami, Z. , Sarkhani, N. , & Nikpeyma, N. (2022). Decision fatigue in nurses in the COVID‐19 pandemic: A commentary. Nursing Open, 9(1), 4–5. 10.1002/nop2.1069 34546010 PMC8662001

[nop21614-bib-0031] Hossain, M. R. , Patwary, M. M. , Sultana, R. , & Browning, M. H. E. M. (2021). Psychological distress among healthcare professionals during the early stages of the COVID‐19 outbreak in low resource settings: A cross‐sectional study in Bangladesh. Frontiers in Public Health, 9, 701920. 10.3389/fpubh.2021.701920 34858914 PMC8632035

[nop21614-bib-0032] Huang, L. , Lei, W. , Xu, F. , Liu, H. , & Yu, L. (2020). Emotional responses and coping strategies in nurses and nursing students during COVID‐19 outbreak: A comparative study. PLoS One, 15(8), e0237303. 10.1371/journal.pone.0237303 32764825 PMC7413410

[nop21614-bib-0033] Jack, S. M. , Boyle, M. , McKee, C. , Ford‐Gilboe, M. , Wathen, C. N. , Scribano, P. , Davidov, D. , McNaughton, D. , O'Brien, R. , Johnston, C. , Gasbarro, M. , Tanaka, M. , Kimber, M. , Coben, J. , Olds, D. L. , & MacMillan, H. L. (2019). Effect of addition of an intimate partner violence intervention to a nurse home visitation program on maternal quality of life: A randomized clinical trial. JAMA, 321(16), 1576–1585. 10.1001/jama.2019.3211 31012933 PMC6487547

[nop21614-bib-0034] Jiang, Y. , Hu, B. , Tu, B. , & Zhuang, Q. (2021). Late‐onset PTSD and coping strategies for frontline nurses during the COVID‐19 epidemic in China. Nursing Open, 8(6), 3055–3064. 10.1002/nop2.1018 34392610 PMC8441903

[nop21614-bib-0035] Keynejad, R. C. , Hanlon, C. , & Howard, L. M. (2020). Psychological interventions for common mental disorders in women experiencing intimate partner violence in low‐income and middle‐income countries: A systematic review and meta‐analysis. The Lancet Psychiatry, 7(2), 173–190. 10.1016/S2215-0366(19)30510-3 31981539 PMC7029417

[nop21614-bib-0036] Kim, K.‐J. , Yoo, M. S. , & Seo, E. J. (2018). Exploring the influence of nursing work environment and patient safety culture on missed nursing Care in Korea. Asian Nursing Research, 12(2), 121–126. 10.1016/j.anr.2018.04.003 29684580

[nop21614-bib-0037] Kim‐Soon, N. , Abdulmaged, A. I. , Mostafa, S. A. , Mohammed, M. A. , Musbah, F. A. , Ali, R. R. , & Geman, O. (2022). A framework for analyzing the relationships between cancer patient satisfaction, nurse care, patient attitude, and nurse attitude in healthcare systems. Journal of Ambient Intelligence and Humanized Computing, 13(1), 87–104. 10.1007/s12652-020-02888-x

[nop21614-bib-0038] Lee, M. , & Jang, K.‐S. (2019). Nurses' emotions, emotion regulation and emotional exhaustion. International Journal of Organizational Analysis, 27(5), 1409–1421. 10.1108/IJOA-06-2018-1452

[nop21614-bib-0039] McLindon, E. , Humphreys, C. , & Hegarty, K. (2018). “It happens to clinicians too”: An Australian prevalence study of intimate partner and family violence against health professionals. BMC Women's Health, 18(1), 113. 10.1186/s12905-018-0588-y 29940948 PMC6020247

[nop21614-bib-0040] McNett, M. , Masciola, R. , Sievert, D. , & Tucker, S. (2021). Advancing evidence‐based practice through implementation science: Critical contributions of doctor of nursing practice‐ and doctor of philosophy‐prepared nurses. Worldviews on Evidence‐Based Nursing, 18(2), 93–101. 10.1111/wvn.12496 33856116

[nop21614-bib-0041] Mulhall, A. (2003). In the field: Notes on observation in qualitative research: *Observation in qualitative research* . Journal of Advanced Nursing, 41(3), 306–313. 10.1046/j.1365-2648.2003.02514.x 12581118

[nop21614-bib-0042] Munawar, K. , & Choudhry, F. R. (2021). Exploring stress coping strategies of frontline emergency health workers dealing COVID‐19 in Pakistan: A qualitative inquiry. American Journal of Infection Control, 49(3), 286–292. 10.1016/j.ajic.2020.06.214 32649990 PMC7340021

[nop21614-bib-0043] Nashwan, A. J. , Abujaber, A. A. , Mohamed, A. S. , Villar, R. C. , & Al‐Jabry, M. M. (2021). Nurses' willingness to work with COVID‐19 patients: The role of knowledge and attitude. Nursing Open, 8(2), 695–701. 10.1002/nop2.674 33570275 PMC7877123

[nop21614-bib-0044] Ogbe, E. , Harmon, S. , Van den Bergh, R. , & Degomme, O. (2020). A systematic review of intimate partner violence interventions focused on improving social support and/ mental health outcomes of survivors. PLoS One, 15(6), e0235177. 10.1371/journal.pone.0235177 32584910 PMC7316294

[nop21614-bib-0045] Othman, N. , & Nasurdin, A. M. (2019). Job characteristics and staying engaged in work of nurses: Empirical evidence from Malaysia. International Journal of Nursing Sciences, 6(4), 432–438. 10.1016/j.ijnss.2019.09.010 31728397 PMC6839340

[nop21614-bib-0046] Özçevik Subaşi, D. , Akça Sümengen, A. , Şimşek, E. , & Ocakçı, A. F. (2021). Healthcare workers' anxieties and coping strategies during the COVID‐19 pandemic in Turkey. Perspectives in Psychiatric Care, 57(4), 1820–1828. 10.1111/ppc.12755 33650693 PMC8013878

[nop21614-bib-0047] Pajakoski, E. , Rannikko, S. , Leino‐Kilpi, H. , & Numminen, O. (2021). Moral courage in nursing – An integrative literature review. Nursing & Health Sciences, 23(3), 570–585. 10.1111/nhs.12805 33389792

[nop21614-bib-0048] Pinho, L. , Correia, T. , Sampaio, F. , Sequeira, C. , Teixeira, L. , Lopes, M. , & Fonseca, C. (2021). The use of mental health promotion strategies by nurses to reduce anxiety, stress, and depression during the COVID‐19 outbreak: A prospective cohort study. Environmental Research, 195, 110828. 10.1016/j.envres.2021.110828 33548294 PMC7857980

[nop21614-bib-0049] Rasool, S. F. , Wang, M. , Tang, M. , Saeed, A. , & Iqbal, J. (2021). How toxic workplace environment effects the employee engagement: The mediating role of organizational support and employee wellbeing. International Journal of Environmental Research and Public Health, 18(5), 2294. 10.3390/ijerph18052294 33652564 PMC7956351

[nop21614-bib-0050] Rathnayake, S. , Dasanayake, D. , Maithreepala, S. D. , Ekanayake, R. , & Basnayake, P. L. (2021). Nurses' perspectives of taking care of patients with coronavirus disease 2019: A phenomenological study. PLoS One, 16(9), e0257064. 10.1371/journal.pone.0257064 34478482 PMC8415609

[nop21614-bib-0051] Rayner, G. , Blackburn, J. , Edward, K. , Stephenson, J. , & Ousey, K. (2019). Emergency department nurse's attitudes towards patients who self‐harm: A meta‐analysis. International Journal of Mental Health Nursing, 28(1), 40–53. 10.1111/inm.12550 30387232

[nop21614-bib-0052] Registered Nursing . (2022). Religious and Spiritual Influences on Health: NCLEX‐RN. Retrieved from https://www.registerednursing.org/nclex/religious‐spiritual‐influences‐health/

[nop21614-bib-0053] Richards, O. K. , Iott, B. E. , Toscos, T. R. , Pater, J. A. , Wagner, S. R. , & Veinot, T. C. (2022). “It's a mess sometimes”: Patient perspectives on provider responses to healthcare costs, and how informatics interventions can help support cost‐sensitive care decisions. Journal of the American Medical Informatics Association, 29(6), 1029‐1039. 10.1093/jamia/ocac010 35182148 PMC9093030

[nop21614-bib-0054] Rony, M. K. K. , Bala, S. D. , Rahman, M. M. , Dola, A. J. , Kayesh, I. , Islam, M. T. , Tama, I. J. , Shafi, E. H. , & Rahman, S. (2021). Experiences of front‐line nurses caring for patients with COVID‐19 in Bangladesh: A qualitative study. Belitung Nursing Journal, 7(5), 380‐386. 10.33546/bnj.1680 PMC10367993 37496501

[nop21614-bib-0055] Rony, M. K. K. , Islam, K. , & Alamgir, H. M. (2022). Coping strategies that motivated frontline nurses while caring for the COVID‐19 patients during the pandemic: A scoping review. Journal of Nursing Management, 30(6), 1881–1891. 10.1111/jonm.13644 35483749 PMC9115125

[nop21614-bib-0056] Sapri, N. D. , Ng, Y. T. , Wu, V. X. , & Klainin‐Yobas, P. (2022). Effectiveness of educational interventions on evidence‐based practice for nurses in clinical settings: A systematic review and meta‐analysis. Nurse Education Today, 111, 105295. 10.1016/j.nedt.2022.105295 35144204

[nop21614-bib-0057] Sarkar, P. , Debnath, N. , & Reang, D. (2021). Coupled human‐environment system amid COVID‐19 crisis: A conceptual model to understand the nexus. Science of the Total Environment, 753, 141757. 10.1016/j.scitotenv.2020.141757 32891990 PMC7434356

[nop21614-bib-0058] Siddiqui, K. (2020). Bangladesh suffers from 76 percent shortage of nurses. Retrieved from https://tbsnews.net/bangladesh/health/bangladesh‐suffers‐76‐percent‐shortage‐nurses‐76387

[nop21614-bib-0060] Suandika, M. , Tang, W.‐R. , Ulfah, M. , & Cahyaningrum, E. D. (2021). Self‐confidence of nurses philosophy: A concept analysis. Open Access Macedonian Journal of Medical Sciences, 9(T4), 206–211. 10.3889/oamjms.2021.5788

[nop21614-bib-0061] Sundborg, E. , Törnkvist, L. , Wändell, P. , & Saleh‐Stattin, N. (2018). Impact of an educational intervention for district nurses about preparedness to encounter women exposed to intimate partner violence. Scandinavian Journal of Caring Sciences, 32(2), 902–913. 10.1111/scs.12521 28922452

[nop21614-bib-0062] Tong, A. , Sainsbury, P. , & Craig, J. (2007). Consolidated criteria for reporting qualitative research (COREQ): A 32‐item checklist for interviews and focus groups. International Journal for Quality in Health Care, 19(6), 349–357. 10.1093/intqhc/mzm042 17872937

[nop21614-bib-0063] Usman, M. , & Fahy, S. (2021). Coping with the COVID‐19 crisis: An overview of service adaptation and challenges encountered by a rural psychiatry of later life (POLL) team. Irish Journal of Psychological Medicine, 38(4), 288–292. 10.1017/ipm.2020.86 32611473 PMC7399143

[nop21614-bib-0064] Weare, R. , Green, C. , Olasoji, M. , & Plummer, V. (2019). ICU nurses feel unprepared to care for patients with mental illness: A survey of nurses' attitudes, knowledge, and skills. Intensive and Critical Care Nursing, 53, 37–42. 10.1016/j.iccn.2019.03.001 30878535

[nop21614-bib-0065] Wolff, M. B. , O'Connor, P. J. , Wilson, M. G. , & Gay, J. L. (2021). Associations between occupational and leisure‐time physical activity with employee stress, burnout and well‐being among healthcare industry workers. American Journal of Health Promotion, 35(7), 957–965. 10.1177/08901171211011372 34105386

[nop21614-bib-0066] World Health Organization . (2021). Health and Care Worker Deaths during COVID‐19. Retrieved from https://www.who.int/news/item/20‐10‐2021‐health‐and‐care‐worker‐deaths‐during‐covid‐19

[nop21614-bib-0067] Worldometer . (2022). COVID‐19 CORONAVIRUS PANDEMIC. Retrieved from https://www.worldometers.info/coronavirus/

[nop21614-bib-0068] Yektatalab, S. , Momennasab, M. , Parvizy, S. , & Mousazadeh, N. (2022). Improving Nurses' job satisfaction: An action research study. Systemic Practice and Action Research, 35(1), 15–32. 10.1007/s11213-021-09554-z

[nop21614-bib-0069] Zhang, M. , Niu, N. , Zhi, X. , Zhu, P. , Wu, B. , Wu, B. , Meng, A. , & Zhao, Y. (2021). Nurses' psychological changes and coping strategies during home isolation for the 2019 novel coronavirus in China: A qualitative study. Journal of Advanced Nursing, 77(1), 308–317. 10.1111/jan.14572 33068024

[nop21614-bib-0070] Zhang, X. , Jiang, X. , Ni, P. , Li, H. , Li, C. , Zhou, Q. , Ou, Z. , Guo, Y. , & Cao, J. (2021). Association between resilience and burnout of front‐line nurses at the peak of the COVID‐19 pandemic: Positive and negative affect as mediators in Wuhan. International Journal of Mental Health Nursing, 30(4), 939–954. 10.1111/inm.12847 33893718 PMC8251287

[nop21614-bib-0071] Zhu, H. , Liu, X. , Yao, L. , Zhou, L. , Qin, J. , Zhu, C. , Ye, Z. , & Pan, H. (2022). Workplace violence in primary hospitals and associated risk factors: A cross‐sectional study. Nursing Open, 9(1), 513–518. 10.1002/nop2.1090 34655279 PMC8685843

